# Identifying on admission patients likely to develop acute kidney injury in hospital

**DOI:** 10.1186/s12882-019-1237-x

**Published:** 2019-02-14

**Authors:** Anastasios Argyropoulos, Stuart Townley, Paul M. Upton, Stephen Dickinson, Adam S. Pollard

**Affiliations:** 1 Centre for Implementation Science, Faculty of Health Sciences, University of Southampton, Southampton, SO17 1BJ UK; 2 College of Engineering, Mathematics, and Physical Sciences, University of Exeter, Penryn, Cornwall, TR10 9FE UK; 3 Research, Development, and Innovation, Royal Cornwall Hospitals NHS Trust, Truro, TR1 3HD UK

**Keywords:** Acute kidney injury, AKI, Fuzzy logic, Multivariable logistic regression, Risk factors, Forward selection

## Abstract

**Background:**

The incidence of Acute Kidney Injury (AKI) continues to increase in the UK, with associated mortality rates remaining significant. Approximately one fifth of hospital admissions are associated with AKI and approximately a third of patients with AKI in hospital develop AKI during their time in hospital. A fifth of these cases are considered avoidable. Early risk detection remains key to decreasing AKI in hospitals, where sub-optimal care was noted for half of patients who developed AKI.

**Methods:**

Electronic anonymised data for adults admitted into the Royal Cornwall Hospitals Trust (RCHT) between 18th March and 31st December 2015 was trimmed to that collected within the first 24 h of hospitalisation. These datasets were split according to three separate time periods: data used for training the Takagi-Sugeno Fuzzy Logic Systems (FLS) and the multivariable logistic regression (MLR) models; data used for testing; and data from a later patient spell used for validation.

Three fuzzy logic models and three MLR models were developed to link characteristics of patients diagnosed with a maximum stage AKI within 7 days of admission: the first models to identify any AKI Stage (FLS I, MLR I), the second for patterns of AKI Stage 2 or 3 (FLS II, MLR II), and the third to identify AKI Stage 3 (FLS III, MLR III). Model accuracy is expressed by area under the curve (AUC).

**Results:**

Accuracy for each model during internal validation was: FLS I and MLR I (AUC 0.70, 95% CI: 0.64–0.77); FLS II (AUC 0.77, 95% CI: 0.69–0.85) and MLR II (AUC 0.74, 95% CI: 0.65–0.83); FLS III and MLR III (AUC 0.95, 95% CI: 0.92–0.98).

**Conclusions:**

FLS II and FLS III (and the respective MLR models) can identify with a high level of accuracy patients at high risk of developing AKI in hospital. These two models cannot be properly assessed against prior studies as this is the first attempt at quantifying the risk of developing specific Stages of AKI for a broad cohort of both medical and surgical inpatients. FLS I and MLR I performance is comparable to other existing models.

**Electronic supplementary material:**

The online version of this article (10.1186/s12882-019-1237-x) contains supplementary material, which is available to authorized users.

## Background

Acute Kidney Injury (AKI) is emerging as an important clinical syndrome which is associated with poor clinical outcomes. AKI, previously known as Acute Renal Failure, refers to a sudden reduction in kidney function.

The Kidney Disease: Improving Global Outcomes (KDIGO) Acute Kidney Injury Work Group definitions of AKI have been adopted in the UK. This classification identifies three stages of severity of AKI, based on rises in serum creatinine or reduction in urine output (Table 1) [1, 2]. At the Royal Cornwall Hospitals Trust (RCHT) clinicians predominantly use creatinine change more often than urine output to classify AKI Stage, and in this study, we only used creatinine change.

**Table 1:** Measurements used to classify the three Stages of AKI

Stage	Serum Creatinine	Urine output
1	1.5–1.9 x baseline creatinine, or ≥ 26.4 μmol/l within 48 h	< 0.5 ml/kg/hr. for 6–12 h
2	2.0–2.9 x baseline creatinine	< 0.5 ml/kg/hr. for ≥12 h
3	≥ 3.0 times the baseline, or ≥ 354 μmol/l, or initiation of renal replacement therapy	< 0.3 ml/kg/hr. for ≥24 h, or anuria ≥12 h

The majority of AKI is caused by sepsis and hypovolaemia. Other causes include renal tract obstruction and intrinsic renal disease. Around 1 in 5 adult hospital admissions are associated with AKI [3]. AKI is present on admission in around 60% of all patients with AKI detected in hospital [4]. AKI is associated with high mortality rates; from 8 to 18%, 22–33%, and 32–36% mortality for patients with AKI Stages 1, 2, and 3 respectively, whilst in the absence of AKI, mortality runs at 2% [4–6]. Consequently, in England AKI may be associated with as many as 40,000 excess deaths each year [6]. Hospital inpatient AKI care has been estimated to cost £1 billion each year, and effective prevention of AKI could save up to £200 million per annum [6].

The 2009 National Confidential Enquiry into Patient Outcome and Death (NCEPOD) AKI report found that care could have been improved for 50% of patients [7]. Since the NCEPOD report, great strides have been made in hospital AKI care, including the introduction in UK hospitals of an AKI algorithm to detect AKI and national advice on what an AKI Bundle should include [2, 8]. Clinically recognised risk factors for AKI include modifiable factors (e.g. use of iodinated contrast, use of certain medications) and non-modifiable factors (e.g. age, the presence of chronic kidney disease) [9]; combinations of these modifiable and non-modifiable factors appear to multiply risks of developing AKI. Roberts et al. describe that age alone is closely associated with the number of AKI risk factors patients have, as well as itself being associated with AKI [10].

Given that approximately a third of patients with AKI in hospital develop AKI during their stay in hospital, two-thirds of patients with AKI in hospital have AKI at the time of admission [4]. So, there is an opportunity to prevent the development of AKI in a large number of patients: in cases of AKI developed whilst in hospital, 20% are avoidable [7]. An AKI risk prediction tool could identify patients at high risk of AKI and provide the attending medical teams the opportunity to prevent the development of AKI by intervening in patients’ current care. For example, staff would be alerted to the risk faced by individual patients, prompting a review of the patient’s current clinical condition, and current medication, which may lead to increased clinical monitoring or a change in treatment. This could cut rates of AKI, leading to a reduction in morbidity, mortality, and length of stay.

We describe an attempt at predicting which patients will go on to develop AKI in hospital, and specifically which Stage of AKI. This is undertaken using methods based around fuzzy logic which are then calibrated against more conventional approaches.

## Methods

Three fuzzy logic systems (FLS) (FLS I, II, III) and three multivariable logistic regression (MLR) models (MLR I, II, III) were constructed to stratify patient risk for developing AKI (any Stage), either Stage 2 or 3 AKI, or Stage 3 AKI respectively during a stay in hospital. Each model was parameterised with data from a time period and validated using data from a later period. Data was provided by RCHT. Each FLS was distinguishable from the other due to the different sets of risk factors used in defining FLS I, II and III respectively.

### Data collection

Research presented here draws on data relating to patients admitted into RCHT in the UK (www.royalcornwall.nhs.uk). RCHT serves roughly 430,000 people and employs about 5000 staff with a budget of approximately £380 million. Across its three sites it admits approximately 59,000 emergencies (including maternity and births) and 9000 elective inpatients per year into 750 beds.

Construction of a FLS involves three stages: training, testing, and validation. Each FLS was trained using anonymised data for 5504 patients treated by RCHT between 18/03/2015 and 30/09/2015; tested using equivalent data for 937 patients from October 2015; and validated with RCHT data for 1020 patients treated during November and December 2015. The same process was followed for the development and validation of the MLR models.

### Risk factor definition and initial identification

AKI was identified using serum creatinine levels only through the usual laboratory method at the hospital, i.e. the NHS England AKI algorithm identified the stage of AKI on all blood samples received in the laboratory [2]. The NHS England AKI algorithm is based on the KDIGO AKI classification (Table 1).

Risk factors for use in developing the models were selected following clinical discussion and literature review. The list of risk factors was reduced by variables not defined by data availability electronically for all inpatients. Consequently, 25 variables were initially selected for entry into each model.

For risk factors: platelets, white blood count (WBC), red blood count (RBC), haematocrit (HCT), haemoglobin (Hb), mean platelet volume (MPV), mean corpuscular volume (MCV), sodium (Na), potassium (K), chloride (CL-), urea, creatinine and albumin, data records available between 24 h before admission and 24 h after admission for patients undergoing tests in that time window were kept. If the data records were available for both the day before admission and the day of admission, the record from the day before admission was selected. Given the absence of the exact processing time of test results, where multiple of the same type were undertaken per patient per day, a result was selected at random from the test of the same type performed. The remaining risk factors were available on admission, which were, for each patient:

- Age (years), gender (male or female), type of admission (medical or surgical)
- Prescription of non-steroidal anti-inflammatory drugs (NSAID), prescription of angiotensin converting enzyme inhibitors (ACEI)
- Comorbidities: diabetes, heart failure, chronic kidney disease (CKD) and vascular disease (VD) of coronary artery and heart, not including acute myocardial infarction and associated complications. Patients with these comorbidities were identified as those presenting with known pre-existing conditions.

Data were excluded from this study for patients under 18 years of age, for patients staying less than 48 h in hospital and for patients who developed AKI > 7 days after admission. Incomplete records were also removed.

### Statistical analysis

The association between dependent and independent variables aiming to identify the most suitable risk factors to be included in each model was examined by performing univariable and multivariable analyses using SPSS version 24.0. Initially, univariable logistic regression analysis was conducted to determine the odds ratios of AKI incidence for each risk factor. Next, multiple logistic regression models based on forward stepwise selection were developed by including risk factors with univariable *p*-values< 0.25. A value of *p* ≤ 0.05 was considered to be statistically significant.

### Fuzzy logic system development

Takagi-Sugeno-Kang (TSK) or Sugeno fuzzy logic (FL) models based on subtractive clustering were developed using the Fuzzy Logic Toolbox within the framework of Matlab R2016a. The TSK FLS methodology was originally developed as an approach to represent a series of systems and functions by utilising input-output data [11, 12]. The absence of a systematic way to derive appropriate parameters for the FLS can be overcome with data clustering. Further details regarding the design of TSK FLS can be found in [13].The fuzzy modelling algorithm is portrayed in Additional file 1: Figure S1.

### Model performance measures

The Area Under the Curve (AUC) was calculated for each Receiver Operating Characteristic (ROC) Curve to evaluate the effectiveness of the models [14]. Model performance was also evaluated using sensitivity (SN) and specificity (SP) with a single decision threshold determined for each model during testing and applied during the validation process based on the method that minimises the absolute value of Specificity-Sensitivity [15]. Analysis was performed using R (3.4.3) with packages “OptimalCutpoints” and “pROC” utilised in the calculation of the optimal values and performances [16, 17].

To assess whether any loss in model accuracy might be caused by the use of TSK FL, equivalent models for FLS I, II and III were assembled using Logistic Regression [15]. Logistic Regression has been widely applied in developing risk assessment frameworks in healthcare, and as such, is adept at “ground truthing” performance of each of the FLS models [18].

## Results

### Patient characteristics

A summary of the characteristics of patients with and without AKI is shown in Additional file 1: Table S1. Of 5504 patient admissions into RCHT, 216 (3.9%) were diagnosed with any Stage AKI during hospitalisation and were included in the development of FLS I (training). For FLS I testing, the equivalent patient numbers were 937 and 89 (9.5%); and for FLS I validation, 1020 and 65 (6.4%) respectively. In general, AKI patients were older than those without AKI (mean (SD) age was 75.1 (14.2) years vs 66.7 (19.4) years), had a higher incidence of heart failure (30.3% vs 13.5%) and CKD (34.6% vs 13.5%) and a lower incidence of NSAID (2.7% vs 7.0%) when compared to low risk patients.

Additional file 1: Table S3 has the equivalent patient characteristics for data feeding FLS II, with 94 (1.7%) of 5504 patients contracting Stage 2 AKI or Stage 3 AKI used in training FLS II. For FLS II testing and validation, respectively 30 (3.2%) of 937 patients and 28 (2.7%) of 1020 patients developed Stage 2 AKI or Stage 3 AKI. Stage 2 AKI and Stage 3 AKI patients were older (age was 74.7 (14.6) years vs 67.0 (19.4)) and were more likely to have heart failure (28.3% vs 14%), diabetes (27.6% vs 19%) and CKD (38.8% vs 14.1%) when compared to low risk patients or patients who developed Stage 1 AKI.

Patient characteristics of patients at low AKI risk, patients diagnosed with Stage 1 AKI or Stage 2 AKI, along with patients who developed Stage 3 AKI are depicted in Additional file 1: Table S4. Thirty- six records (0.65%) of patients who contracted Stage 3 AKI were used in the design of FLS III (training); 16 (1.7%) diagnosed with Stage 3 AKI were used in FLS III calibration process (testing) and 14 (1.4%) in the validation. Stage 3 AKI patients were older (age was 72.8 (15.4) years vs 67.1 (19.3)) and had a higher heart failure (25.8% vs 14.2%) and CKD (45.5% vs 14.3%) prevalence when compared to low risk patients or patients who developed Stage 1 AKI or Stage 2 AKI.

Biochemical marker distributions and clinical characteristics are summarised in Additional file 1: Table S1, Table S2, Table S3 and Table S4.

### Risk factor selection

#### FLS I and MLR I

Univariable analysis aimed at identifying risk factors to for inclusion in FLS I (Table 2) revealed that platelets (*p* = 0.041), RBC (*p* < 0.001), HTC (*p* = 0.001), Haemoglobin (p < 0.001), MPV (p < 0.001), MCV (*p* = 0.095), Sodium (*p* = 0.034), Potassium (p < 0.001), Chloride (*p* = 0.069), Urea (p < 0.001), Creatinine (p < 0.001), Albumin (p < 0.001), age (p < 0.001), gender (*p* = 0.027), NSAID (*p* = 0.009), ACEI (*p* = 0.037), diabetes (p = 0.009), heart failure (p < 0.001), CKD (p < 0.001) and VD (of coronary artery and heart not including acute myocardial infarction and associated complications) (*p* = 0.006) were associated with the development of any Stage AKI. Despite the fact that the *p* value of the type of admission (*p* = 0.803 > 0.25) did not fulfil the selection criteria, it was considered by the authors that it could serve as a complementary predictor despite the univariable prefiltering [19]. Especially in the context of multiple regression, complementary predictors can be found that when used separately are unable to explain differences between classes (i.e. are not significant), but together they are [20]. This proved to be the case for the type of admission in the risk factor identification. Following forward selection, MPV (*p* = 0.033), Urea (*p* = 0.007), Albumin (*p* < 0.001), age (p < 0.001), type of admission (*p* = 0.018), heart failure (p < 0.001) and CKD (*p* = 0.013) were retained for model development (Table 2).

**Table 2:** Risk factors associated with AKI (any stage) by univariable and multivariable analysis

Factors	Univariable Analysis			Multivariable Analysis		
	OR	95% CI	*p*-Value	OR	95% CI	*p*-Value
Platelets (10^a^9/L)	0.999	0.997- 1.000	0.041			
WBC (10^a^9/L)	1.006	0.988- 1.025	0.487			
RBC (10^12^/L)	0.707	0.589–0.848	< 0.001			
HCT (L/L)	0.030	0.004–0.236	0.001			
Hb (g/dL)	0.988	0.982–0.993	< 0.001			
MPV (fL)	1.181	1.079–1.294	< 0.001	1.109	1.008–1.221	0.033
MCV (fL)	1.017	0.997–1.036	0.095			
Na (mmol/L)	0.973	0.949–0.998	0.034			
K (mmol/L)	1.482	1.193–1.841	< 0.001			
CL- (mmol/L)	0.980	0.958–1.002	0.069			
Urea (mmol/L)	1.066	1.048–1.084	< 0.001	1.028	1.008–1.049	0.007
Creatinine (mmol/L)	1.002	1.001–1.003	< 0.001			
Albumin (g/L)	0.936	0.916–0.955	< 0.001	0.953	0.931–0.976	< 0.001
Age (years)	1.035	1.025–1.045	< 0.001	1.023	1.013–1.034	< 0.001
Gender	1.015	1.002–1.029	0.027			
Chronic_OP (days)	0.999	0.991–1.007	0.716			
Chronic_IP (days)	1.060	0.807–1.392	0.674			
Admission type	0.964	0.722–1.286	0.803	1.440	1.064–1.949	0.018
NSAID	0.303	0.124–0.741	0.009			
ACEI	1.445	1.023–2.041	0.037			
Diabetes	1.514	1.108–2.068	0.009			
Heart Failure	2.920	2.168–3.932	< 0.001	1.905	1.378–2.633	< 0.001
CKD	3.117	2.319–4.189	< 0.001	1.560	1.099–2.213	0.013
VD^a^	1.523	1.131- 2.052	0.006			

^a^of coronary artery and heart, not including acute myocardial infarction and associated complications

#### FLS II and MLR II

Univariable analysis showed that RBC (*p* = 0.076), HCT (*p* = 0.066), Haemoglobin (*p* = 0.009), MPV (*p* = 0.008), Potassium (*p* < 0.001), Urea (p < 0.001), Creatinine (p < 0.001), Albumin (p < 0.001), age (p < 0.001), NSAID (*p* = 0.152), ACEI (*p* = 0.201), diabetes (*p* = 0.017), heart failure (*p* = 0.001), CKD (*p* < 0.001) and VD (of coronary artery and heart not including acute myocardial infarction and associated complications) (*p* = 0.214) predicted patients diagnosed with Stage 2 or Stage 3 AKI (Table 3). As explained above, the type of admission (*p* = 0.673) was once again included in the multivariable analysis. However, following forward selection, Urea (*p* < 0.001), Albumin (*p* = 0.002) and age (*p* = 0.016) were identified as the most suitable risk factors (Table 3).

**Table 3:** Risk factors associated with AKI (Stage 2 or Stage 3) by univariable and multivariable analysis

Factors	Univariable Analysis			Multivariable Analysis		
	OR	95% CI	*p*-Value	OR	95% CI	*p*-Value
Platelets (10^a^9/L)	0.999	0.997- 1.001	0.414			
WBC (10^a^9/L)	1.005	0.978- 1.033	0.718			
RBC (10^12^/L)	0.780	0.592–1.026	0.076			
HCT (L/L)	0.054	0.002–1.212	0.066			
Hb (g/dL)	0.988	0.980–0.997	0.009			
MPV (fL)	1.174	1.044–1.321	0.008			
MCV (fL)	1.002	0.973–1.032	0.887			
Na (mmol/L)	0.996	0.957–1.036	0.842			
K (mmol/L)	1.854	1.372–2.507	< 0.001			
CL- (mmol/L)	0.994	0.961–1.029	0.740			
Urea (mmol/L)	1.071	1.048–1.094	< 0.001	1.055	1.032–1.079	< 0.001
Creatinine (mmol/L)	1.003	1.002–1.004	< 0.001			
Albumin (g/L)	0.932	0.904–0.961	< 0.001	0.948	0.917–0.981	0.002
Age (years)	1.027	1.014–1.041	< 0.001	1.017	1.003–1.032	0.016
Gender	0.965	0.641–1.453	0.865			
Chronic_OP (days)	1.001	0.977–1.026	0.940			
Chronic_IP (days)	0.967	0.912–1.026	0.268			
Admission type	1.096	0.717–1.675	0.673			
NSAID	0.430	0.136–1.365	0.152			
ACEI	1.404	0.835–2.360	0.201			
Diabetes	1.730	1.101–2.719	0.017			
Heart Failure	2.210	1.390–3.515	0.001			
CKD	2.789	1.788–4.350	< 0.001			
VD^a^	1.336	0.846- 2.108	0.214			

^a^of coronary artery and heart, not including acute myocardial infarction and associated complications

#### FLS III and MLR III

MPV (*p* = 0.006), Potassium (*p* < 0.001), Chloride (*p* = 0.15), Urea (p < 0.001), Creatinine (p < 0.001), Albumin (*p* = 0.011) and CKD (p = 0.002) were identified as potential predictors of Stage 3 AKI using univariable analysis (Table 4). Similarly to the process followed in the risk factor selection for multivariable analysis for FLS I and FLS II, age (*p* = 0.261) and type of admission (*p* = 0.340) were added to the variables identified by the univariable analysis. Following forward selection, Chloride (*p* = 0.001), Creatinine (p < 0.001) and Albumin (*p* = 0.023) were included in the model (Table 4).

**Table 4:** Risk factors associated with Stage 3 AKI by univariable and multivariable analysis

Factors	Univariable Analysis			Multivariable Analysis		
	OR	95% CI	*p*-Value	OR	95% CI	*p*-Value
Platelets (10^a^9/L)	0.998	0.995- 1.001	0.264			
WBC (10^a^9/L)	1.004	0.959- 1.051	0.874			
RBC (10^12^/L)	1.041	0.659–1.645	0.864			
HCT (L/L)	2.352	0.012–444.759	0.749			
Hb (g/dL)	1.001	0.986–1.016	0.941			
MPV (fL)	1.222	1.060–1.408	0.006			
MCV (fL)	1.004	0.958–1.053	0.856			
Na (mmol/L)	0.976	0.919–1.037	0.429			
K (mmol/L)	2.246	1.449–3.481	< 0.001			
CL- (mmol/L)	0.964	0.916–1.013	0.150	0.968	0.949–0.987	0.001
Urea (mmol/L)	1.064	1.034–1.094	< 0.001			
Creatinine (mmol/L)	1.003	1.002–1.005	< 0.001	1.003	1.002–1.004	< 0.001
Albumin (g/L)	0.937	0.892–0.985	0.011	0.945	0.899–0.992	0.023
Age (years)	1.011	0.992–1.030	0.261			
Gender	1.375	0.711–2.659	0.344			
Chronic_OP (days)	1.010	0.976–1.045	0.573			
Chronic_IP (days)	0.981	0.909–1.058	0.612			
Admission type	1.382	0.711–2.687	0.340			
NSAID	0.000	0.000–0.000	0.994			
ACEI	0.735	0.259–2.083	0.562			
Diabetes	1.215	0.552–2.674	0.628			
Heart Failure	0.967	0.375–2.493	0.944			
CKD	3.083	1.535–6.191	0.002			
VD^a^	1.159	0.544- 2.472	0.702			

^a^of coronary artery and heart, not including acute myocardial infarction and associated complications

### Model development and performance

By applying the design process depicted in the Additional file 1: (Figure S1), for each model scope the FLS with the best performance during the testing process was identified using subtractive clustering. For FLS I optimal performance was reached when each input had two fuzzy sets based on the clustering performed and two rules governed the fuzzy system’s function (fuzzy model). FLS II performed best when each input had three fuzzy sets based on the clustering performed and three rules governed the fuzzy system’s function (fuzzy model). FLS III performance was maximised when each input had four fuzzy sets based on the clustering performed and four rules governed the fuzzy system’s function (fuzzy model).

The FLSs’ membership function parameters, rules and consequent parameters as well as the MLR models’ coefficients, that were derived using the training dataset, are provided in Additional file 1: Table S5- Table S8.

### Model interpretation

**Fig. 1:**
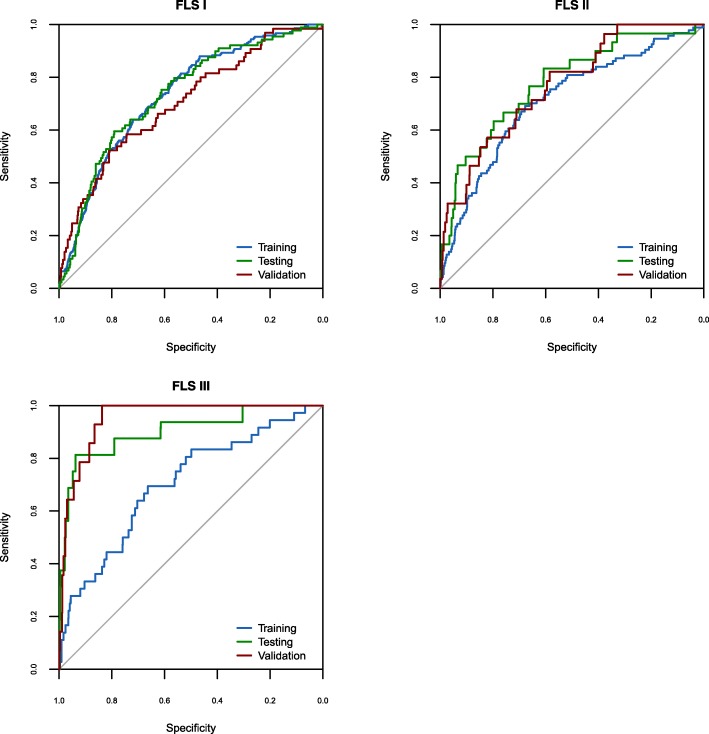
FLS I, FLS II, FLS III Receiver operating characteristic (ROC) curves for training, testing and validation

**Fig. 2:**
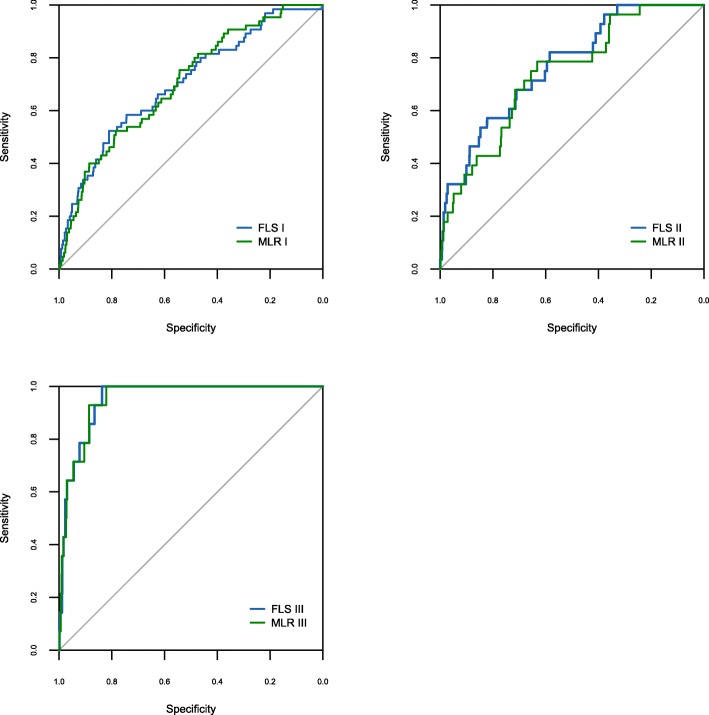
FLS and MLR validation Receiver operating characteristic (ROC) curves

The ROC curves and their corresponding AUC values were calculated for each stage (training, testing, and validation) of each FLS (FLS I, II, and III) (Fig. 1) and of each MLR (MLR I, II, III) to demonstrate the ability of each model to discriminate between low risk and high risk AKI patients (Table 5). Comparative performance between each FLS and its respective MLR is shown in Table 5, Table 6 and Fig. 2.

**Table 5:** AUC values with corresponding 95% confidence intervals for all the models

Model	Model Performance					
	Training^a^		Testing^a^		Validation^a^	
	AUC	95% CI	AUC	95% CI	AUC	95% CI
FLS I	0.74	0.71–0.77	0.73	0.68–0.79	0.70	0.64–0.77
MLR I	0.73	0.70–0.77	0.73	0.68–0.78	0.70	0.64–0.77
FLS II	0.75	0.70–0.79	0.78	0.69–0.87	0.77	0.69–0.85
MLR II	0.71	0.66–0.77	0.78	0.69–0.87	0.74	0.65–0.83
FLS III	0.79	0.71–0.86	0.90	0.81–0.99	0.95	0.92–0.98
MLR III	0.70	0.61–0.79	0.82	0.68–0.96	0.95	0.92–0.98

^a^When calculating AUC for all models, a significant p value resulted (*p* < 0**.**0001) during training, testing, and validation

**Table 6:** Sensitivity and specificity for all the models

Model	Cut-off	Testing				Validation			
		SN	SP	PPV	NPV	SN	SP	PPV	NPV
FLS I	4.34×10^−2^	0.63	0.64	0.17	0.95	0.62	0.64	0.1	0.96
MLR I	4.2×10^−2^	0.67	0.69	0.18	0.99	0.57	0.69	0.11	0.96
FLS II	1.905×10^−2^	0.7	0.7	0.07	0.99	0.61	0.73	0.06	0.99
MLR II	1.95×10^−2^	0.7	0.7	0.07	0.99	0.61	0.72	0.06	0.99
FLS III	8.019×10^−3^	0.81	0.81	0.07	1.0	1.0	0.83	0.08	1.0
MLR III	7.376×10^−3^	0.75	0.75	0.05	0.99	1.0	0.79	0.06	1.0

For FLS I, AUC during validation was 0.7 (95% CI: 0.64–0.77) with SN and SP holding the values of 0.62 and 0.64 respectively. Positive predictive value (PPV) 0.1 and negative predictive value (NPV) was 0.96 (Table 5). Performance differed in MLR I only for SN and SP generated which were 0.57 and 0.69 respectively (Table 6).

In the case of FLS II, AUC (validation) was 0.77 (95% CI: 0.69–0.85), SN = 0.61, SP = 0.73, PPV = 0.06 and NPV = 0.99. For the case of MLR II, the AUC dropped to 0.74 (95% CI: 0.65–0.83) versus FLS II whilst SP changed slightly to 0.72 (Table 5, Table 6).

Model FLS III yielded an AUC during validation of 0.95 (95% CI: 0.92–0.98), producing a sensitivity of 1.0 and a specificity of 0.83, with PPV = 0.08 and NPV = 1.0. Although the AUC generated by MLR III mirrored that of FLS III, values of SP and PPV dropped to 0.79 and 0.06 respectively (Table 5, Table 6).

## Discussion

AKI is categorised into three Stages according to severity. The KDIGO stages of AKI are identified using creatinine measurements from blood tests, or from the reduction in urine output. Other methods to identify AKI such as using novel biomarkers are not used in practice, are evolving, and so were not analysed as part of this study [21]. Current practice only assesses the likelihood of developing AKI (in stages 1, 2, or 3) and does not offer discriminated risk assessment, especially for more dangerous stages of AKI later during their hospital spell, including Stage 2 AKI (22–33% mortality) and Stage 3 AKI (32–36% mortality). This discriminated capability is provided by our risk assessment model through the use of a fresh set of risk factors for each stage of AKI.

Each patient carries a risk of developing AKI at any time during hospitalisation. Research presented here quantifies that risk using information obtained at the point of admission and within the patient’s first two days in hospital. Additionally, the capability to assess the risk of acquiring the more severe strands of acute kidney injury has never previously been attempted using a TSK FLS.

According to this research, use of FLS III could quickly have identified, within the first two days of a hospital spell, all 14 patients who went on to develop Stage 3 AKI during their stay. Moreover, with a specificity of 83%, use of the same model would have falsely identified as “high risk” 17% of patients carrying low risk.

Susceptibility to either Stage 2 or Stage 3 is assessed by FLS II which correctly classifies 61% of high-risk patients and 73% of patients at low risk. Model performance for forecasting the development of any Stage of AKI, using FLS I, was marginally worse, predicting 62% of cases that develop in RCHT and 64% of people not developing the disease.

The purpose behind development of FLS I was to forge comparison with risk tools previously assembled and, thereby assess the suitability of the selected input parameter set in predicting the patients at risk of developing AKI. Therefore, using the same model development technique but with either a refined or an expanded input parameter set, including prospectively collected data, enhanced predictive accuracy might be achieved.

FLS II and FLS III model in particular cannot be properly assessed against prior studies as this is the first attempt at quantifying the risk of developing specific Stages of AKI for a broad cohort of both medical and surgical inpatients. So, we believe our study has generated unrivalled accuracy in identifying patients at risk of Stage 3 AKI (AUC = 0·95, 95% CI: 0·92–0·92). For Stage 2 or 3 AKI, FLS II model performance dropped comparatively (AUC = 0·77, 95% CI: 0·69–0·85), to a level lower than the AUC = 0.9 (95% CI: 0.9–0.9) and AUC = 0.87 (95% CI: 0.87–0.87) resulting from the application of a Gradient Boosting Machine algorithm forecasting patients developing Stage 2 AKI within 24 and 48 h respectively [22]. However, the approach presented here identifies patients at elevated risk of AKI up to a week ahead, allowing for a longer period of mitigating measures to be implemented. Likewise, a week is longer than the forecasting window employed in other research which used ward-monitored serum creatinine to predict onset of each Stage of AKI over the following 24 h [23]. Despite this shorter window, the AUC of 0.83 (95% CI: 0.83–0.84) for Stage 3 AKI underperformed FLS III.

Another attempt at stratifying the risk of AKI development in-hospital used electronic patient data gathered 24 h either side of admission. Although categories employed to portray disease severity do not match AKI Stages used in this research and in clinical practice, an AUC of 0.75 (95% CI: 0.73–0.76) resulted [24]. In the case of FLS I the accuracy of our approach fell slightly further (AUC = 0·70, 95% CI: 0·64–0·77). However, the AUC for FLS I is comparable with a validation of an AKI Prediction score which was applied to identify development of hospital-acquired AKI in general medical and surgical admissions; for medical patients with no known baseline serum creatinine an AUC of up to 0.71 was achieved [25]. This contrasts to a number of studies which have looked to assess the risk of AKI in medical admissions. In analysing 898 patient records Roberts et al. generated AUC values of approximately 0·7 [10]. Marginally better levels of model performance were obtained by a study in Sussex, UK (AUC = 0·72) [26]. Similarly, a study from a hospital in Kent, UK processed data from non-maternity, emergency admissions only and predicted the onset of AKI on admission (AUC = 0.75) and 72 h post admission (AUC = 0·68) [27].

In addition to forecasting AKI up to a week prior to development, our study utilises data obtained 24 h either side of admission, thereby reducing computational burden. This contrasts to an approach which uses data gathered across varying windows, up to 30 days prior to admission [28]. Although the AUC reached 0.765, 0.73 and 0.7 (to predict AKI at times 1, 2 and 3 days prior respectively), patients in this cohort were limited to between 18 and 64 years old, and the researchers did not explore the applicability of the models for the elderly.

Our study performs less well than that employing up to five modelling techniques using data collected within the first 24 h of a hospital stay from patients aged 60 or over (AUC = 0.74, 95% CI: 0.73–0.76) [29]. However, in addition to the age restriction, the research used variables which were not available electronically for all patient admissions at RCHT, e.g. BMI and Family History.

FLS I, however, has comparable performance to all of these studies, and uses data from both medical and surgical patients and both emergency and non-emergency.

According to our study data, mortality from AKI across our study period stood at 38% for Stage 3 and 17% for both AKI Stage 1 and AKI Stage 2. Given approximately 30% of cases are considered preventable, we propose that use of an accurate risk assessment tool for the development of in-hospital AKI Stage 3 could save lives. Based on the results presented in this study, the use of FLS III could evolve the potential to avoid some of these deaths.

The ability of FLS III to predict patients at low risk of AKI Stage 3 is not as good as it is for those at high risk. Falsely mitigating against some conditions, e.g. Deep Vein Thrombosis, can bring about unacceptable risks, i.e. bleeds. However, we consider the potential harm to the patient from inappropriate mitigation is likely to be sufficiently outweighed by the marginal risk caused by withholding measures. So, the use of a tool such as FLS III is worthy of health economic testing for the prevention of AKI Stage 3 in hospital.

A model of mitigation would include informing the patient’s doctor, pharmacist, and nurse of their elevated risk. This would then bring forward a reassessment of the patient that includes:

- Reviewing their current condition and current diagnosis;
- considering alternative causes of illness (particularly searching for signs of sepsis);
- reviewing dates of recent and/or planned iodinated contrast scans;
- reviewing their current medication (particularly medication with an effect on blood pressure and renal haemo-dynamics);
- examining their fluid status and assessing the need for IV fluids [30]; and
- planning future blood tests and physiological observation monitoring.

These measures might be considered low-cost and add minimal risk for patients to whom they are unnecessarily introduced, including those falsely identified as being at high risk. The specificity results of this study indicate that mitigating measures will be unnecessarily introduced to roughly a third of patients carrying low risk who are expected to stay more than two days in hospital. In addition, given the lack of intrusiveness as these measures are introduced to patients, this is likely to be considered acceptable.

The performance of each FLS was assessed against MLR, a methodology more traditionally employed in developing risk assessment frameworks in healthcare. In all three models, the performance of FLS was at least as good as MLR, perhaps signalling that FLS could be employed more widely in health risk assessment and epidemiological research.

It is hypothesised that by identifying a patient’s risk of developing AKI Stage 3 at the earliest point possible during a patient spell this will increase survival. Based on the data period within this study, the fraction of patients dying in hospital following a diagnosis of Stage 3 AKI during hospitalisation stands at 38% (25 out of 66). Assuming one fifth of these cases were preventable and FLS III would have early identified 80%, use of this risk assessment tool could have prevented approximately 4 deaths during the same hospital stay.

Of 304 patients who went on to develop Stage 1 or Stage 2 AKI, 53 died. Assuming one fifth preventable and a FLS sensitivity of 60%, a further 6 lives might have been saved with early risk assessment.

However, these claims should be tempered because later development of AKI can be a signature of a general end-of-life decline and greater mortality risk [24]. Nevertheless, due to the high levels of predictive accuracy achieved by research and given that 20% of cases of AKI developed in hospital are avoidable, it offers considerable potential to cut the in-hospital burden of the disease if accompanied by appropriate mitigating measures.

## Conclusions

Acute Kidney Injury can develop in patients in hospital with the severest type, Stage 3, having a mortality rate of between 30 and 40%. With appropriate mitigation, some cases can be avoided. We have used anonymised data taken from patients as they were admitted to hospital to create risk assessment models using Fuzzy Logic and multivariable logistic regression. The models were trained to recognise patterns in data and distinguish between those patients who went onto develop AKI any Stage, AKI Stage 2 and 3, or AKI Stage 3 and those who did not develop AKI at all. This was the first attempt at building separate models for the three currently recognised degrees of AKI severity and upon testing the models, our ability to predict AKI Stage 3 was very strong, albeit on a small number of cases. Subsequently, our view is given that AKI is preventable in some cases and any technology containing these models is designed to be deployed early on in a patient’s stay, it has the potential to save lives.

## Additional file

Additional file 1: The additional file contains: 1) Flow diagram of the fuzzy modelling algorithm, 2) additional information regarding patient characteristics, 3) FLS membership function parameters, rules and consequent parameters, 4) MLR models’. (DOCX 74 kb)

## Abbreviations

ACEI: Angiotensin converting enzyme inhibitors
AKI: Acute Kidney Injury
AUC: Area under the curve
CKD: Chronic kidney disease
FL: Fuzzy logic
FLS: Fuzzy logic system
Hb: Haemoglobin
HCT: Haematocrit
KDIGO: Kidney Disease Improving Global Outcomes
MCV: Mean corpuscular volume
MLR: Multivariable logistic regression
MPV: Mean platelet volume
NCEPOD: National Confidential Enquiry into Patient Outcome and Death
NPV: Negative predictive value
NSAID: Non-steroidal anti-inflammatory drugs
PPV: Positive predictive value
RBC: Red blood count
RCHT: Royal Cornwall Hospitals NHS Trust
ROC: Receiver operating characteristic
SD: Standard deviation
SN: Sensitivity
SP: Specificity
TSK: Takagi-Sugeno-Kang
VD: Vascular disease
WBC: White blood count
