# Supplementary material for: Identifying on admission patients likely to develop acute kidney injury in hospital
Source: BMC Nephrol. 2019 Feb 14;20:56. doi: 10.1186/s12882-019-1237-x (PMC6376785; doi:10.1186/s12882-019-1237-x)
Supplement: Supplementary file 1 — The additional file contains: 1) Flow diagram of the fuzzy modelling algorithm, 2) additional information regarding patient characteristics, 3) FLS membership function parameters, rules and consequent parameters, 4) MLR models’. (DOCX 74 kb) [file 12882_2019_1237_MOESM1_ESM.docx]

# Identifying on admission patients likely to develop acute kidney injury in hospital

# Additional file 1

## Flow diagram of the fuzzy modelling algorithm

##

Figure S- 1 Sugeno FL system based on subtractive clustering design [1]

##

## Patient characteristics

Table S- 1 Patient characteristics for FLS I (Data are n (%) or mean (SD); median (IQR))

| **Factors** | | **Training** | | **Testing** | | **Validation** | |
| --- | --- | --- | --- | --- | --- | --- | --- |
|  |  | **Healthy**  **(n=5288)** | **AKI Stage 1,2 or 3 (n=216)** | **Healthy**  **(n= 848)** | **AKI Stage 1,2 or 3**  **(n= 89)** | **Healthy**  **(n= 955)** | **AKI Stage 1,2 or 3**  **(n= 65)** |
| **Platelets (10*9/L)** | | 268.1 (122.5);  247.0 (191.0-316.0) | 250.7 (127.8);  223.0 (183.3-294.8) | 277.7 (121.8); 253.0 (203.3-325.8) | 276.0 (116.3); 253.0 (206.5-356.0) | 278.5 (117.6); 256.0 (204.0-329.0) | 283.6 (168.7); 252.0 (169.5-348.0) |
| **WBC (10*9/L)** | | 11.2 (6.5);  10.2 (7.6-13.6) | 11.5 (5.3);  10.5 (8.0-13.9) | 11.1 (5.4);  10.2 (7.8-13.3) | 14.3 (19.4);  11.5 (8.5-14.8) | 10.9 (5.2);  9.8 (7.3-13.5) | 11.3 (5.6);  10.4 (7.3-13.8) |
| **RBC (10^12^/L)** | | 4.3 (0.7);  4.3 (3.8-4.7) | 4.1 (0.8);  4.1 (3.7-4.6) | 4.3 (0.7);  4.3 (3.9-4.7) | 4.2 (0.8);  4.2 (3.7-4.7) | 4.3 (0.7);  4.4 (3.9-4.8) | 4.1 (0.8);  4.1 (3.5-4.6) |
| **HCT (L/L)** | | 0.4 (0.1);  0.4 (0.4-0.4) | 0.4 (0.1);  0.4 (0.3-0.4) | 0.4 (0.1);  0.4 (0.4-0.4) | 0.4 (0.1);  0.4 (0.3-0.4) | 0.4 (0.1);  0.4 (0.4-0.4) | 0.4 (0.1);  0.4 (0.3-0.4) |
| **Hb (g/dL)** | | 126.3 (21.9);  128.0 (113.0-141.0) | 119.9 (22.6);  121.0 (106.0-134.0) | 127.8 (20.4);  128.0 (115.3-142.0) | 124.5 (23.6);  127.0 (106.5-141.0) | 127.8 (21.5);  130.0 (114.0-143.0) | 120.5 (23.8);  123.0 (107.5-136.5) |
| **MPV (fL)** | | 8.2 (1.1);  8.1 (7.5-8.7) | 8.5 (1.3);  8.2 (7.7-9.0) | 8.3 (1.0);  8.2 (7.6-8.9) | 8.4 (0.9);  8.3 (7.8-9.1) | 8.4 (1.0);  8.3 (7.7-8.9) | 8.6 (1.0);  8.5 (7.9-9.3) |
| **MCV (fL)** | | 92.0 (6.9);  91.7 (87.8-96.0) | 92.8 (7.6);  92.5 (88.4-96.4) | 92.4 (6.9);  92.0 (88.4-96.3) | 93.2 (9.4);  92.0 (88.4-97.3) | 92.1 (6.9);  92.1 (88.0-96.0) | 93.3 (8.0);  93.5 (87.9-98.0) |
| **Na (mmol/L)** | | 136.9 (5.1);  138.0 (134.0-140.0) | 136.2 (6.0);  137.0 (133.0-139.8) | 136.9 (4.6);  137.0 (135.0-140.0) | 134.5 (7.6);  136.0 (132.0-139.0) | 137.1 (4.8);  138.0 (135.0-140.0) | 135.0 (6.8);  136.0 (133.0-140.0) |
| **K (mmol/L)** | | 4.3 (0.6);  4.3 (3.9-4.6) | 4.4 (0.8);  4.3 (4.0-4.8) | 4.3 (0.5);  4.2 (3.9-4.6) | 4.3 (0.7);  4.4 (3.9-4.8) | 4.2 (0.6);  4.2 (3.9-4.5) | 4.6 (1.0);  4.4 (4.1-5.0) |
| **CL- (mmol/L)** | | 97.1 (5.8);  98.0 (94.0-101.0) | 96.4 (6.8);  97.0 (92.0-101.0) | 97.3 (5.2);  98.0 (94.3-101.0) | 94.8 (7.9);  95.0 (91.0-100.0) | 98.1 (5.5);  99.0 (95.0-102.0) | 96.1 (7.0);  98.0 (92.5-100.5) |
| **Urea (mmol/L)** | | 7.2 (5.2);  5.9 (4.2-8.3) | 10.5 (7.3);  8.5 (6.1-12.9) | 6.2 (4.0);  5.2 (3.8-7.2) | 9.1 (6.8);  8.0 (5.0-11.3) | 6.3 (4.5);  5.1 (3.8-7.2) | 10.4 (8.6);  7.0 (4.7-14.7) |
| **Creatinine (mmol/L)** | | 97.6 (84.0);  78.0 (63.0-101.0) | 128.9 (82.6);  106.0 (81.0-154.3) | 89.3 (58.1);  77.0 (63.0-98.0) | 142.4 (115.7); 108.0 (74.0-156.0) | 90.0 (62.4);  76.0 (60.0-98.0) | 184.2 (180.9); 120.0 (77.5-209.5) |
| **Albumin (g/L)** | | 39.8 (5.9);  40.0 (36.0-44.0) | 37.3 (6.4);  38.0 (33.0-42.0) | 39.5 (5.7);  40.0 (36.0-43.0) | 35.9 (7.2);  37.0 (31.0-40.5) | 39.1 (5.6);  40.0 (35.0-43.0) | 37.1 (6.1);  38.0 (33.0-41.0) |
| **Age** | | 66.7 (19.4);  71.0 (56.0-82.0) | 76.4 (14.3);  80.0 (69.3-87.0) | 67.3 (19.5);  71.0 (55.0-83.0) | 74.1 (13.9);  76.0 (68.0-83.0) | 66.5 (19.8);  71.0 (54.0-82.0) | 72.1 (13.9);  74.0 (63.5-83.0) |
| **Gender** | **Male** | 2770 (52.4%) | 110 (50.9%) | 445 (52.5%) | 40 (44.9%) | 488 (51.1%) | 38 (58.5%) |
|  | **Female** | 2518 (47.6%) | 106 (49.1%) | 403 (47.5%) | 49 (55.1%) | 467 (48.9%) | 27 (41.5%) |
| **Chronic_OP (days)** | | 5.6 (8.2);  3.0 (0.0-8.0) | 6.9 (9.4);  4.0 (0.0-10.0) | 5.6 (8.9);  3.0 (0.0-8.0) | 6.6 (9.6);  4.0 (0.0-10.0) | 5.4 (7.9);  3.0 (0.0-7.0) | 6.8 (8.4);  4.0 (0.0-10.0) |
| **Chronic_IP (days)** | | 3.8 (20.4);  1.0 (0.0-3.0) | 3.2 (20.0);  1.0 (0.0-2.0) | 2.5 (10.9);  1.0 (0.0-2.0) | 11.0 (52.0);  0.0 (0.0-2.0) | 3.0 (19.1);  1.0 (0.0-2.0) | 17.4 (63.8);  1.0 (0.0-3.0) |
| **Admission type** | **Medical** | 3482 (65.8%) | 144 (66.7%) | 533 (62.9%) | 57 (64.0%) | 608 (63.7%) | 38 (58.5%) |
|  | **Surgical** | 1806 (34.2%) | 72 (33.3%) | 315 (37.1%) | 32 (36.0%) | 347 (36.3%) | 27 (41.5%) |
| **NSAID** | **No** | 4905 (92.8%) | 211 (97.7%) | 784 (92.5%) | 85 (95.5%) | 904 (94.7%) | 64 (98.5%) |
|  | **Yes** | 383 (7.2%) | 5 (2.3%) | 64 (7.5%) | 4 (4.5%) | 51 (5.3%) | 1 (1.5%) |
| **ACEI** | **No** | 4531 (85.7%) | 174 (80.6%) | 736 (86.8%) | 77 (86.5%) | 873 (91.4%) | 56 (86.2%) |
|  | **Yes** | 757 (14.3%) | 42 (19.4%) | 112 (13.2%) | 12 (13.5%) | 82 (8.6%) | 9 (13.8%) |
| **Diabetes** | **No** | 4295 (81.2%) | 160 (74.1%) | 684 (80.7%) | 62 (69.7%) | 773 (80.9%) | 53 (81.5%) |
|  | **Yes** | 993 (18.8%) | 56 (25.9%) | 164 (19.3%) | 27 (30.3%) | 182 (19.1%) | 12 (18.5%) |
| **Heart Failure** | **No** | 4569 (86.4%) | 148 (68.5%) | 741 (87.4%) | 61 (68.5%) | 826 (86.5%) | 49 (75.4%) |
|  | **Yes** | 719 (13.6%) | 68 (31.5%) | 107 (12.6%) | 28 (31.5%) | 129 (13.5%) | 16 (24.6%) |
| **CKD** | **No** | 4583 (86.7%) | 146 (67.6%) | 736 (86.8%) | 58 (65.2%) | 812 (85.0%) | 38 (58.5%) |
|  | **Yes** | 705 (13.3%) | 70 (32.4%) | 112 (13.2%) | 31 (34.8%) | 143 (15.0%) | 27 (41.5%) |
| **VD*** | **No** | 4123 (78.0%) | 151 (69.9%) | 656 (77.4%) | 62 (69.7%) | 745 (78.0%) | 54 (83.1%) |
|  | **Yes** | 1165 (22.0%) | 65 (30.1%) | 192 (22.6%) | 27 (30.3%) | 210 (22.0%) | 1. 16.9%) |

**of coronary artery and heart, not including acute myocardial infarction and associated complications*

Table S- 2 Total patient characteristics (training, testing and validation) for FLS I, FLS II and FLS III. (Data are n (%) or mean (SD); median (IQR))

| **Factors** | | **FLS I** | | **FLS II** | | **FLS III** | |
| --- | --- | --- | --- | --- | --- | --- | --- |
|  |  | **Healthy patients**  **(n=7091)** | **AKI Stage 1,2 or 3 (n=370)** | **Healthy patients or AKI Stage 1**  **(n= 7309)** | **AKI Stage 2 or 3**  **(n= 152)** | **Healthy patients or AKI Stage 1 or 2**  **(n= 7395)** | **AKI Stage 3**  **(n= 66)** |
| **Platelets (10*9/L)** | | 270.6 (121.8);  249.0 (194.0-319.0) | 262.6 (133.7); 232.0 (183.0-315.8) | 270.4 (122.2); 249.0 (194.0-319.0) | 265.1 (131.8); 237.0 (185.0-320.0) | 270.4 (122.6); 249.0 (194.0-319.0) | 256.1 (104.8); 242.5 (177.5-343.0) |
| **WBC (10*9/L)** | | 11.2 (6.2);  10.1 (7.6-13.5) | 12.2 (10.6);  10.6 (7.9-14.0) | 11.2 (6.2);  10.1 (7.6-13.6) | 12.9 (13.8);  10.6 (7.7-14.8) | 11.2 (6.5);  10.1 (7.6-13.6) | 11.6 (5.7);  10.6 (7.5-14.6) |
| **RBC (10^12^/L)** | | 4.3 (0.7);  4.3 (3.8-4.7) | 4.1 (0.8);  4.1 (3.6-4.6) | 4.3 (0.7);  4.3 (3.8-4.7) | 4.1 (0.8);  4.1 (3.6-4.6) | 4.3 (0.7);  4.3 (3.8-4.7) | 4.1 (0.9);  3.9 (3.6-4.6) |
| **HCT (L/L)** | | 0.4 (0.1);  0.4 (0.4-0.4) | 0.4 (0.1);  0.4 (0.3-0.4) | 0.4 (0.1);  0.4 (0.4-0.4) | 0.4 (0.1);  0.4 (0.3-0.4) | 0.4 (0.1);  0.4 (0.4-0.4) | 0.4 (0.1);  0.4 (0.3-0.4) |
| **Hb (g/dL)** | | 126.7 (21.7);  128.0 (113.0-142.0) | 121.1 (23.1);  122.5 (106.0-136.0) | 126.5 (21.7);  128.0 (113.0-142.0) | 120.5 (23.9);  121.0 (105.0-134.8) | 126.4 (21.8);  128.0 (113.0-141.0) | 121.0 (25.7);  121.5 (102.0-136.3) |
| **MPV (fL)** | | 8.2 (1.0);  8.1 (7.6-8.7) | 8.5 (1.1);  8.3 (7.7-9.1) | 8.3 (1.0);  8.1 (7.6-8.7) | 8.6 (1.2);  8.4 (7.9-9.1) | 8.3 (1.0);  8.1 (7.6-8.7) | 8.7 (1.4);  8.4 (7.9-9.2) |
| **MCV (fL)** | | 92.1 (6.9);  91.8 (87.9-96.0) | 93.0 (8.1);  92.7 (88.3-96.8) | 92.1 (6.9);  91.9 (87.9-96.1) | 93.0 (7.4);  92.8 (88.2-96.4) | 92.1 (6.9);  91.9 (87.9-96.0) | 94.1 (7.3);  93.4 (88.1-97.9) |
| **Na (mmol/L)** | | 136.9 (5.0);  138.0 (134.0-140.0) | 135.6 (6.6);  137.0 (133.0-139.3) | 136.9 (5.0);  138.0 (134.0-140.0) | 135.8 (6.1);  136.0 (133.0-140.0) | 136.9 (5.1);  138.0 (134.0-140.0) | 135.6 (5.6);  136.0 (133.8-139.0) |
| **K (mmol/L)** | | 4.3 (0.6);  4.2 (3.9-4.6) | 4.4 (0.8);  4.4 (4.0-4.8) | 4.3 (0.6);  4.2 (3.9-4.6) | 4.6 (0.9);  4.4 (4.0-5.1) | 4.3 (0.6);  4.3 (3.9-4.6) | 4.6 (0.9);  4.5 (4.0-5.2) |
| **CL-(mmol/L)** | | 97.3 (5.7);  98.0 (94.0-101.0) | 96.0 (7.1);  97.0 (92.0-100.0) | 97.3 (5.8);  98.0 (94.0-101.0) | 95.9 (6.6);  96.0 (91.3-100.0) | 97.2 (5.8);  98.0 (94.0-101.0) | 95.4 (5.9);  95.0 (91.0-98.3) |
| **Urea (mmol/L)** | | 7.0 (5.0);  5.7 (4.1-8.1) | 10.1 (7.5);  8.1 (5.5-12.7) | 7.1 (5.0);  5.8 (4.1-8.1) | 11.9 (9.5);  9.2 (5.7-14.1) | 7.1 (5.1);  5.8 (4.1-8.2) | 12.1 (8.5);  8.9 (6.2-15.6) |
| **Creatinine (mmol/L)** | | 95.6 (78.8);  78.0 (62.0-100.0) | 141.8 (115.1); 108.5 (78.0-157.3) | 96.2 (78.7);  78.0 (62.0-101.0) | 176.6 (150.8); 124.5 (83.3-207.8) | 96.8 (79.4);  78.0 (63.0-102.0) | 220.1 (177.3); 163.0 (105.3-263.3) |
| **Albumin (g/L)** | | 39.7 (5.8);  40.0 (36.0-44.0) | 36.9 (6.6);  38.0 (33.0-41.0) | 39.6 (5.8);  40.0 (36.0-44.0) | 36.2 (7.0);  36.5 (31.0-41.0) | 39.6 (5.9);  40.0 (36.0-44.0) | 35.1 (7.7);  36.0 (29.0-40.0) |
| **Age** | | 66.7 (19.4);  71.0 (55.0-82.0) | 75.1 (14.2);  77.5 (67.8-86.0) | 67.0 (19.4);  71.0 (56.0-82.0) | 74.7 (14.6);  77.0 (66.0-86.0) | 67.1 (19.3);  71.0 (56.0-82.0) | 72.8 (15.4);  75.0 (65.0-83.0) |
| **Gender** | **Male** | 3703 (52.2%) | 188 (50.8%) | 3809 (52.1%) | 82 (53.9%) | 3865 (52.3%) | 26 (39.4%) |
|  | **Female** | 3388 (47.8%) | 182 (49.2%) | 3500 (47.9%) | 70 (46.1%) | 3530 (47.7%) | 40 (60.6%) |
| **Chronic_OP (days)** | | 5.6 (8.3);  3.0 (0.0-8.0) | 6.8 (9.3);  4.0 (0.0-10.0) | 5.6 (8.3);  3.0 (0.0-8.0) | 6.7 (8.4); 3.0 (0.0-10.0) | 5.6 (8.3);  3.0 (0.0-8.0) | 8.9 (9.9);  7.0 (0.0-14.3) |
| **Chronic_IP (days)** | | 3.5 (19.3);  1.0 (0.0-2.0) | 7.6 (40.2);  1.0 (0.0-2.0) | 3.5 (19.3);  1.0 (0.0-2.0) | 13.9 (57.5); 1.0 (0.0-2.0) | 3.5 (19.2);  1.0 (0.0-2.0) | 30.1 (84.9);  1.0 (0.0-4.0) |
| **Admission type** | **Medical** | 4623 (65.2%) | 239 (64.6%) | 4763 (65.2%) | 99 (65.1%) | 4821 (65.2%) | 41 (62.1%) |
|  | **Surgical** | 2468 (34.8%) | 131 (35.4%) | 2546 (34.8%) | 53 (34.9%) | 2574 (34.8%) | 25 (37.9%) |
| **NSAID** | **No** | 6593 (93.0%) | 360 (97.3%) | 6805 (93.1%) | 148 (97.4%) | 6887 (93.1%) | 66 (100.0%) |
|  | **Yes** | 498 (7.0%) | 10 (2.7%) | 504 (6.9%) | 4 (2.6%) | 508 (6.9%) | 0 (0.0%) |
| **ACEI** | **No** | 6140 (86.6%) | 307 (83.0%) | 6321 (86.5%) | 126 (82.9%) | 6388 (86.4%) | 59 (89.4%) |
|  | **Yes** | 951 (13.4%) | 63 (17.0%) | 988 (13.5%) | 26 (17.1%) | 1007 (13.6%) | 7 (10.6%) |
| **Diabetes** | **No** | 5752 (81.1%) | 275 (74.3%) | 5917 (81.0%) | 110 (72.4%) | 5975 (80.8%) | 52 (78.8%) |
|  | **Yes** | 1339 (18.9%) | 95 (25.7%) | 1392 (19.0%) | 42 (27.6%) | 1420 (19.2%) | 14 (21.2%) |
| **Heart Failure** | **No** | 6136 (86.5%) | 258 (69.7%) | 6285 (86.0%) | 109 (71.7%) | 6345 (85.8%) | 49 (74.2%) |
|  | **Yes** | 955 (13.5%) | 112 (30.3%) | 1024 (14.0%) | 43 (28.3%) | 1050 (14.2%) | 17 (25.8%) |
| **CKD** | **No** | 6131 (86.5%) | 242 (65.4%) | 6280 (85.9%) | 93 (61.2%) | 6337 (85.7%) | 36 (54.5%) |
|  | **Yes** | 960 (13.5%) | 128 (34.6%) | 1029 (14.1%) | 59 (38.8%) | 1058 (14.3%) | 30 (45.5%) |
| **VD*** | **No** | 5524 (77.9%) | 267 (72.2%) | 5677 (77.7%) | 114 (75.0%) | 5741 (77.6%) | 50 (75.8%) |
|  | **Yes** | 1567 (22.1%) | 103 (27.8%) | 1632 (22.3%) | 38 (25.0%) | 1654 (22.4%) | 16 (24.2%) |

**of coronary artery and heart, not including acute myocardial infarction and associated complications*

Table S- 3 Patient characteristics for FLS II (Data are n (%) or mean (SD); median (IQR))

| **Factors** | | **Training** | | **Testing** | | **Validation** | |
| --- | --- | --- | --- | --- | --- | --- | --- |
|  |  | **Healthy patients or AKI Stage 1**  **(n= 5410)** | **AKI Stage 2 or 3**  **(n= 94)** | **Healthy patients or AKI Stage 1**  **(n= 907)** | **AKI Stage 2 or 3**  **(n= 30)** | **Healthy patients or AKI Stage 1**  **(n= 992)** | **AKI Stage 2 or 3**  **(n= 28)** |
| **Platelets (10*9/L)** | | 267.6 (122.8);  246.0 (190.8-316.0) | 257.2 (116.0); 232.0 (186.5-307.8) | 278.4 (121.9); 253.0 (204.0-328.0) | 252.8 (95.6);  232.5 (184.0-321.3) | 278.1 (118.6); 256.0 (203.0-328.5) | 304.9 (197.2); 254.5 (165.0-387.0) |
| **WBC (10*9/L)** | | 11.2 (6.4);  10.2 (7.6-13.6) | 11.5 (5.4);  10.5 (7.8-14.5) | 11.2 (6.0);  10.2 (7.8-13.3) | 17.6 (28.8);  12.8 (8.0-15.6) | 10.9 (5.2);  9.9 (7.2-13.5) | 12.4 (6.9);  9.8 (7.4-18.5) |
| **RBC (10^12^/L)** | | 4.2 (0.7);  4.3 (3.8-4.7) | 4.1 (0.8);  4.1 (3.7-4.5) | 4.3 (0.7);  4.3 (3.9-4.7) | 4.1 (0.9);  4.1 (3.6-4.8) | 4.3 (0.7);  4.4 (3.9-4.8) | 4.0 (0.9);  4.1 (3.5-4.6) |
| **HCT (L/L)** | | 0.4 (0.1);  0.4 (0.4-0.4) | 0.4 (0.1);  0.4 (0.3-0.4) | 0.4 (0.1);  0.4 (0.4-0.4) | 0.4 (0.1);  0.4 (0.3-0.5) | 0.4 (0.1);  0.4 (0.4-0.4) | 0.4 (0.1);  0.4 (0.3-0.4) |
| **Hb (g/dL)** | | 126.1 (21.9);  128.0 (113.0-141.0) | 120.2 (24.0);  121.0 (105.0-134.0) | 127.6 (20.6);  128.0 (115.0-142.0) | 124.5 (23.2);  124.5 (104.5-142.3) | 127.6 (21.6);  130.0 (114.0-143.0) | 117.4 (24.7);  119.5 (98.3-132.8) |
| **MPV (fL)** | | 8.2 (1.1);  8.1 (7.5-8.7) | 8.5 (1.4);  8.2 (7.7-9.0) | 8.3 (1.0);  8.2 (7.6-8.9) | 8.8 (0.7);  8.6 (8.3-9.1) | 8.4 (1.0);  8.3 (7.7-8.9) | 8.6 (0.9);  8.5 (8.1-9.3) |
| **MCV (fL)** | | 92.0 (6.9);  91.8 (87.8-96.0) | 92.1 (6.6);  91.7 (87.9-96.1) | 92.4 (7.2);  91.9 (88.3-96.3) | 94.5 (8.1);  93.0 (90.2-97.5) | 92.1 (6.9);  92.1 (88.0-96.1) | 94.1 (8.7);  93.9 (87.9-97.6) |
| **Na (mmol/L)** | | 136.9 (5.1);  138.0 (134.0-140.0) | 136.8 (5.8);  138.0 (133.0-141.0) | 136.7 (4.9);  137.0 (134.0-140.0) | 133.8 (6.5);  134.0 (130.8-138.0) | 137.0 (4.9);  138.0 (134.0-140.0) | 134.8 (6.4);  135.5 (133.0-139.0) |
| **K (mmol/L)** | | 4.3 (0.6);  4.3 (3.9-4.6) | 4.5 (0.9);  4.5 (4.0-5.1) | 4.3 (0.6);  4.2 (3.9-4.6) | 4.3 (0.8);  4.3 (3.8-4.8) | 4.2 (0.6);  4.2 (3.9-4.6) | 4.9 (1.2);  4.5 (4.1-5.3) |
| **CL- (mmol/L)** | | 97.1 (5.8);  98.0 (94.0-101.0) | 96.9 (6.6);  97.0 (92.0-101.3) | 97.2 (5.5);  98.0 (94.0-101.0) | 94.0 (6.8);  94.5 (91.0-98.0) | 98.1 (5.5);  99.0 (95.0-102.0) | 94.6 (6.2);  96.0 (91.0-99.0) |
| **Urea (mmol/L)** | | 7.3 (5.2);  5.9 (4.3-8.4) | 11.6 (8.9);  9.3 (6.1-13.6) | 6.3 (4.1);  5.3 (3.8-7.5) | 11.6 (9.9);  9.0 (5.6-13.8) | 6.4 (4.6);  5.2 (3.8-7.3) | 13.0 (10.9);  8.6 (5.3-18.7) |
| **Creatinine (mmol/L)** | | 98.0 (83.6);  79.0 (63.0-102.0) | 145.7 (104.8); 109.5 (77.3-179.3) | 91.1 (60.7);  78.0 (63.0-101.0) | 191.8 (148.1); 141.5 (85.3-259.5) | 91.3 (64.1);  76.0 (61.0-99.0) | 264.0 (233.1); 162.0 (85.8-430.3) |
| **Albumin (g/L)** | | 39.8 (5.9);  40.0 (36.0-44.0) | 37.0 (6.7);  38.0 (32.0-42.0) | 39.3 (5.9);  40.0 (36.0-43.0) | 33.8 (7.3);  35.5 (27.0-39.0) | 39.1 (5.5);  40.0 (35.0-43.0) | 36.1 (7.5);  37.0 (31.5-39.5) |
| **Age** | | 66.9 (19.3);  71.0 (56.0-82.0) | 75.0 (15.2);  77.0 (66.0-87.0) | 67.7 (19.3);  71.0 (56.0-83.0) | 75.2 (13.2);  78.0 (66.8-84.5) | 66.7 (19.6);  71.0 (54.0-82.0) | 73.0 (14.3);  74.5 (58.5-82.8) |
| **Gender** | **Male** | 2830 (52.3%) | 50 (53.2%) | 467 (51.5%) | 18 (60.0%) | 512 (51.6%) | 14 (50.0%) |
|  | **Female** | 2580 (47.7%) | 44 (46.8%) | 440 (48.5%) | 12 (40.0%) | 480 (48.4%) | 14 (50.0%) |
| **Chronic_OP (days)** | | 5.7 (8.3);  3.0 (0.0-8.0) | 5.7 (7.3);  3.0 (0.0-9.0) | 5.7 (9.0);  3.0 (0.0-8.0) | 7.0 (8.7);  4.0 (0.0-11.0) | 5.4 (7.8);  3.0 (0.0-7.0) | 9.6 (10.9);  6.0 (0.3-14.5) |
| **Chronic_IP (days)** | | 3.8 (20.5);  1.0 (0.0-3.0) | 1.7 (2.6);  1.0 (0.0-2.0) | 2.5 (10.6);  1.0 (0.0-2.0) | 29.1 (87.6);  0.0 (0.0-2.0) | 3.0 (18.7);  1.0 (0.0-2.0) | 38.5 (93.9);  2.0 (0.0-7.0) |
| **Admission type** | **Medical** | 3566 (65.9%) | 60 (63.8%) | 567 (62.5%) | 23 (76.7%) | 630 (63.5%) | 16 (57.1%) |
|  | **Surgical** | 1844 (34.1%) | 34 (36.2%) | 340 (37.5%) | 7 (23.3%) | 362 (36.5%) | 12 (42.9%) |
| **NSAID** | **No** | 5025 (92.9%) | 91 (96.8%) | 839 (92.5%) | 30 (100.0%) | 941 (94.9%) | 27 (96.4%) |
|  | **Yes** | 385 (7.1%) | 3 (3.2%) | 68 (7.5%) | 0 (0.0%) | 51 (5.1%) | 1 (3.6%) |
| **ACEI** | **No** | 4629 (85.6%) | 76 (80.9%) | 788 (86.9%) | 25 (83.3%) | 904 (91.1%) | 25 (89.3%) |
|  | **Yes** | 781 (14.4%) | 18 (19.1%) | 119 (13.1%) | 5 (16.7%) | 88 (8.9%) | 3 (10.7%) |
| **Diabetes** | **No** | 4388 (81.1%) | 67 (71.3%) | 723 (79.7%) | 23 (76.7%) | 806 (81.3%) | 20 (71.4%) |
|  | **Yes** | 1022 (18.9%) | 27 (28.7%) | 184 (20.3%) | 7 (23.3%) | 186 (18.8%) | 8 (28.6%) |
| **Heart Failure** | **No** | 4648 (85.9%) | 69 (73.4%) | 784 (86.4%) | 18 (60.0%) | 853 (86.0%) | 22 (78.6%) |
|  | **Yes** | 762 (14.1%) | 25 (26.6%) | 123 (13.6%) | 12 (40.0%) | 139 (14.0%) | 6 (21.4%) |
| **CKD** | **No** | 4664 (86.2%) | 65 (69.1%) | 779 (85.9%) | 15 (50.0%) | 837 (84.4%) | 13 (46.4%) |
|  | **Yes** | 746 (13.8%) | 29 (30.9%) | 128 (14.1%) | 15 (50.0%) | 155 (15.6%) | 15 (53.6%) |
| **VD*** | **No** | 4206 (77.7%) | 68 (72.3%) | 696 (76.7%) | 22 (73.3%) | 775 (78.1%) | 24 (85.7%) |
|  | **Yes** | 1204 (22.3%) | 26 (27.7%) | 211 (23.3%) | 8 (26.7%) | 217 (21.9%) | 4 (14.3%) |

**of coronary artery and heart, not including acute myocardial infarction and associated complications*

Table S- 4 Patient characteristics for FLS III (Data are n (%) or mean (SD); median (IQR))

| **Factors** | | **Training** | | **Testing** | | **Validation** | |
| --- | --- | --- | --- | --- | --- | --- | --- |
|  |  | **Healthy patients or AKI Stage 1 or 2**  **(n= 5468)** | **AKI Stage 3**  **(n= 36)** | **Healthy patients or AKI Stage 1 or 2**  **(n= 921)** | **AKI Stage 3**  **(n= 16)** | **Healthy patients or AKI Stage 1 or 2**  **(n= 1006)** | **AKI Stage 3**  **(n= 14)** |
| **Platelets (10*9/L)** | | 267.6 (122.9);  246.0 (191.0-315.0) | 244.8 (93.9);  246.5 (184.8-316.8) | 277.5 (121.6); 253.0 (203.0-326.5) | 279.6 (99.0);  254.5 (217.3-365.8) | 279.1 (121.2);  256.0 (203.0-329.5) | 258.1 (137.3); 188.0 (156.5-392.0) |
| **WBC (10*9/L)** | | 11.2 (6.4);  10.2 (7.6-13.6) | 11.4 (6.0);  10.1 (7.6-16.0) | 11.4 (8.0);  10.2 (7.8-13.4) | 12.1 (3.8);  12.8 (11.2-14.7) | 10.9 (5.2);  9.9 (7.3-13.5) | 11.5 (7.3);  8.4 (7.1-15.5) |
| **RBC (10^12^/L)** | | 4.2 (0.7);  4.3 (3.8-4.7) | 4.3 (0.9);  4.1 (3.7-4.9) | 4.3 (0.7);  4.3 (3.9-4.7) | 3.9 (0.7);  3.9 (3.5-4.4) | 4.3 (0.7);  4.4 (3.9-4.8) | 3.6 (0.9);  3.6 (3.0-4.6) |
| **HCT (L/L)** | | 0.4 (0.1);  0.4 (0.4-0.4) | 0.4 (0.1);  0.4 (0.3-0.4) | 0.4 (0.1);  0.4 (0.4-0.4) | 0.4 (0.1);  0.4 (0.3-0.4) | 0.4 (0.1);  0.4 (0.4-0.4) | 0.4 (0.1);  0.4 (0.3-0.4) |
| **Hb (g/dL)** | | 126.0 (22.0);  127.0 (112.0-141.0) | 126.3 (27.2);  125.5 (102.5-145.0) | 127.6 (20.7);  128.0 (115.0-142.0) | 118.4 (20.0);  119.0 (103.5-132.5) | 127.5 (21.6);  129.5 (114.0-143.0) | 110.4 (25.7);  117.5 (87.0-132.0) |
| **MPV (fL)** | | 8.2 (1.1);  8.1 (7.5-8.7) | 8.7 (1.8);  8.2 (7.7-9.1) | 8.3 (1.0);  8.2 (7.6-8.9) | 8.8 (0.9);  8.7 (8.2-9.4) | 8.4 (1.0);  8.3 (7.7-8.9) | 8.7 (0.9);  8.7 (7.9-9.3) |
| **MCV (fL)** | | 92.0 (6.9);  91.8 (87.8-96.0) | 92.3 (6.4);  89.8 (87.7-97.1) | 92.4 (7.2);  91.9 (88.4-96.3) | 94.8 (5.9);  93.9 (90.8-98.3) | 92.1 (6.9);  92.1 (88.0-96.1) | 97.9 (9.3);  96.8 (92.4-102.8) |
| **Na (mmol/L)** | | 136.9 (5.1);  138.0 (134.0-140.0) | 136.2 (5.9);  137.5 (134.0-140.8) | 136.7 (5.0); 1  37.0 (134.0-140.0) | 134.4 (4.4);  136.0 (130.3-137.8) | 137.0 (5.0);  138.0 (134.0-140.0) | 135.4 (6.2);  135.5 (133.8-139.0) |
| **K (mmol/L)** | | 4.3 (0.6);  4.3 (3.9-4.6) | 4.6 (0.9);  4.5 (4.0-5.2) | 4.3 (0.6);  4.2 (3.9-4.6) | 4.4 (0.7);  4.4 (4.0-4.9) | 4.2 (0.6);  4.2 (3.9-4.6) | 4.9 (1.1);  4.8 (4.1-5.9) |
| **CL-(mmol/L)** | | 97.1 (5.8);  98.0 (94.0-101.0) | 95.7 (6.8);  95.5 (91.0-101.3) | 97.1 (5.6);  98.0 (94.0-101.0) | 94.5 (4.8);  95.0 (93.3-96.8) | 98.0 (5.6);  99.0 (95.0-102.0) | 95.5 (5.0);  96.5 (91.0-99.0) |
| **Urea (mmol/L)** | | 7.3 (5.3);  5.9 (4.3-8.4) | 11.9 (6.9);  9.9 (6.5-16.6) | 6.4 (4.1);  5.3 (3.8-7.6) | 13.3 (11.9);  8.1 (6.0-15.4) | 6.5 (4.9);  5.2 (3.8-7.3) | 11.2 (8.0);  8.6 (5.6-15.4) |
| **Creatinine (mmol/L)** | | 98.3 (83.5);  79.0 (63.0-102.0) | 181.7 (136.8); 156.5 (90.0-221.8) | 91.9 (61.5);  78.0 (63.0-101.0) | 236.1 (174.4); 183.0 (122.0-280.5) | 93.2 (70.3);  76.0 (61.0-100.0) | 300.6 (245.5); 187.5 (148.0-463.8) |
| **Albumin (g/L)** | | 39.8 (5.9);  40.0 (36.0-44.0) | 37.2 (7.5);  38.5 (31.5-41.8) | 39.2 (5.9);  40.0 (36.0-43.0) | 33.2 (8.4);  35.5 (27.0-38.8) | 39.1 (5.5);  40.0 (35.0-43.0) | 31.7 (6.0);  33.5 (28.5-36.3) |
| **Age** | | 67.1 (19.3);  71.0 (56.0-82.0) | 70.7 (17.2);  75.0 (65.0-80.0) | 67.7 (19.2);  72.0 (56.0-83.0) | 78.6 (9.9);  82.0 (70.5-88.3) | 66.8 (19.5);  71.0 (55.0-82.0) | 71.8 (14.8);  74.5 (57.0-84.0) |
| **Gender** | **Male** | 2864 (52.4%) | 16 (44.4%) | 477 (51.8%) | 8 (50.0%) | 524 (52.1%) | 2 (14.3%) |
|  | **Female** | 2604 (47.6%) | 20 (55.6%) | 444 (48.2%) | 8 (50.0%) | 482 (47.9%) | 12 (85.7%) |
| **Chronic_OP (days)** | | 5.7 (8.3);  3.0 (0.0-8.0) | 6.4 (6.9);  5.5 (0.0-9.8) | 5.7 (9.0);  3.0 (0.0-8.0) | 9.3 (10.8);  5.0 (0.0-18.5) | 5.4 (7.8);  3.0 (0.0-7.0) | 15.0 (12.9);  12.5 (0.0-29.0) |
| **Chronic_IP (days)** | | 3.7 (20.4);  1.0 (0.0-3.0) | 1.9 (3.2);  1.0 (0.0-2.0) | 2.5 (10.5);  1.0 (0.0-2.0) | 53.8 (115.9);  0.0 (0.0-3.3) | 3.0 (18.6);  1.0 (0.0-2.0) | 75.6 (123.9);  7.0 (0.0-145.0) |
| **Admission type** | **Medical** | 3605 (65.9%) | 21 (58.3%) | 577 (62.6%) | 13 (81.3%) | 639 (63.5%) | 7 (50.0%) |
|  | **Surgical** | 1863 (34.1%) | 15 (41.7%) | 344 (37.4%) | 3 (18.8%) | 367 (36.5%) | 7 (50.0%) |
| **NSAID** | **No** | 5080 (92.9%) | 36 (100.0%) | 853 (92.6%) | 16 (100.0%) | 954 (94.8%) | 14 (100.0%) |
|  | **Yes** | 388 (7.1%) | 0 (0.0%) | 68 (7.4%) | 0 (0.0%) | 52 (5.2%) | 0 (0.0%) |
| **ACEI** | **No** | 4673 (85.5%) | 32 (88.9%) | 799 (86.8%) | 14 (87.5%) | 916 (91.1%) | 13 (92.9%) |
|  | **Yes** | 795 (14.5%) | 4 (11.1%) | 122 (13.2%) | 2 (12.5%) | 90 (8.9%) | 1 (7.1%) |
| **Diabetes** | **No** | 4427 (81.0%) | 28 (77.8%) | 733 (79.6%) | 13 (81.3%) | 815 (81.0%) | 11 (78.6%) |
|  | **Yes** | 1041 (19.0%) | 8 (22.2%) | 188 (20.4%) | 3 (18.8%) | 191 (19.0%) | 3 (21.4%) |
| **Heart Failure** | **No** | 4686 (85.7%) | 31 (86.1%) | 795 (86.3%) | 7 (43.8%) | 864 (85.9%) | 11 (78.6%) |
|  | **Yes** | 782 (14.3%) | 5 (13.9%) | 126 (13.7%) | 9 (56.3%) | 142 (14.1%) | 3 (21.4%) |
| **CKD** | **No** | 4705 (86.0%) | 24 (66.7%) | 786 (85.3%) | 8 (50.0%) | 846 (84.1%) | 4 (28.6%) |
|  | **Yes** | 763 (14.0%) | 12 (33.3%) | 135 (14.7%) | 8 (50.0%) | 160 (15.9%) | 10 (71.4%) |
| **VD*** | **No** | 4247 (77.7%) | 27 (75.0%) | 708 (76.9%) | 10 (62.5%) | 786 (78.1%) | 13 (92.9%) |
|  | **Yes** | 1221 (22.3%) | 9 (25.0%) | 213 (23.1%) | 6 (37.5%) | 220 (21.9%) | 1 (7.1%) |

**of coronary artery and heart, not including acute myocardial infarction and associated complications*

## FLS membership function parameters, rules and consequent parameters

Table S- 5 Membership function parameters obtained using subtractive clustering for FLS I

| **Cluster** | **Membership**  **functions** | **Centre; Width(spread)** | | | | | | |
| --- | --- | --- | --- | --- | --- | --- | --- | --- |
|  |  | MPV | Urea | Albumin | Age | Admission Type | Heart failure | CKD |
| 1 | MF 1 | 8; 6.9 | 5.2; 29.68 | 40; 12.77 | 70; 27.37 | 0; 0.30 | 0; 0.30 | 0; 0.30 |
| 2 | MF 2 | 8.3; 6.9 | 6.5; 29.68 | 41; 12.77 | 62; 27.37 | 1; 0.30 | 0; 0.30 | 0; 0.30 |

Table S- 6 Membership function parameters obtained using subtractive clustering for FLS II and FLS III

| **Cluster** | **Membership**  **functions** | **Centre; Width(spread)** | | | | | |
| --- | --- | --- | --- | --- | --- | --- | --- |
|  |  | **FLS II** | | | **FLS III** | | |
|  |  | Urea | Albumin | Age | CL- | Creatinine | Albumin |
| 1 | MF 1 | 7.1; 10 | 40; 4.31 | 77; 9.23 | 98; 5.66 | 86; 143.63 | 41; 3.71 |
| 2 | MF 2 | 5.4; 10 | 43; 4.31 | 54; 9.23 | 96; 5.66 | 90; 143.63 | 32; 3.71 |
| 3 | MF 3 | 4.2; 10 | 45; 4.31 | 28; 9.23 | 101; 5.66 | 78; 143.63 | 49; 3.71 |
| 4 | MF 4 | 8.2; 10 | 30; 4.31 | 83; 9.23 |  |  |  |

Table S- 7 Fuzzy model rules and consequent parameters

| **Models** | **Rule** | **Rule description of the form**  **If … Then y=p_1_·x_1_+ p_2_·x_2_+ …+ p_n_·x_n_+ p_0_** |
| --- | --- | --- |
| **FLS I** | 1 | **If** MPV is MPV_MF 1_ and Urea is Urea_MF 1_ and Albumin is Albumin_MF 1_ and Age is Age _MF 1_ and Admission type is Admission type _MF 1_ and Heart failure is Heart failure _MF 1_ and CKD is CKD _MF 1_ **Then**  Risk= 6·10^-3^· MPV+ 1.8·10^-3^· Urea- 2.2·10^-3^· Albumin+ 2·10^-3^· Age+ 62.0444· Admission type+ 3.34·10^-2^· Heart failure+ 1.82·10^-2^· CKD- 0.2153 |
|  | 2 | **If** MPV is MPV_MF 2_ and Urea is Urea_MF 2_ and Albumin is Albumin_MF 2_ and Age is Age _MF 2_ and Admission type is Admission type _MF 2_ and Heart failure is Heart failure _MF 2_ and CKD is Kindey failure _MF 2_ **Then**  Risk= 6.1·10^-3^· MPV+ 3·10^-3^· Urea- 6.2194·10^-4^· Albumin- 2.3·10^-3^· Age- 29.9376· Admission type+ 4.5·10^-2^· Heart failure+ 4.78·10^-2^· CKD+ 29.7979 |
| **FLS II** | 1 | **If** Urea is Urea_MF 1_ and Albumin is Albumin_MF 1_ and Age is Age _MF 1_ **Then**  Risk= 2.2·10^-3^· Urea- 7.6626·10^-5^· Albumin+ 1.1·10^-3^· Age- 9.17·10^-2^ |
|  | 2 | **If** Urea is Urea_MF 2_ and Albumin is Albumin_MF 2_ and Age is Age _MF 2_ **Then**  Risk= 1.9·10^-3^· Urea- 5.7333·10^-4^· Albumin+ 1.4·10^-3^· Age- 5.01·10^-2^ |
|  | 3 | **If** Urea is Urea_MF 3_ and Albumin is Albumin_MF 3_ and Age is Age _MF 3_ **Then**  Risk= 2.8328·10^-4^· Urea+ 6.7624·10^-4^· Albumin+ 9.6036·10^-4^· Age- 4.89·10^-2^ |
|  | 4 | **If** Urea is Urea_MF 4_ and Albumin is Albumin_MF 4_ and Age is Age _MF 4_ **Then**  Risk= 2.3·10^-3^· Urea- 1.6·10^-3^· Albumin+ 3.4812·10^-5^· Age+ 6.8·10^-2^ |
| **FLS III** | 1 | **If** CL- is CL-_MF 1_ and Creatinine is Creatinine_MF 1_ and Albumin is Albumin _MF 1_ **Then**  Risk= 7.2372·10^-5^· (CL-) – 2.0571·10^-5^· Creatinine - 1.2·10^-3^· Albumin + 4.83·10^-2^ |
|  | 2 | **If** CL- is CL-_MF 2_ and Creatinine is Creatinine_MF 2_ and Albumin is Albumin _MF 2_ **Then**  Risk= 5.0244·10^-4^· (CL-) + 1.112·10^-4^· Creatinine - 1.7·10^-3^· Albumin + 9.92·10^-2^ |
|  | 3 | **If** CL- is CL-_MF 3_ and Creatinine is Creatinine_MF 3_ and Albumin is Albumin _MF 3_ **Then**  Risk= 1.2·10^-3^· (CL-) + 1.9056·10^-4^· Creatinine + 6.8265·10^-4^· Albumin + 7.73·10^-2^ |

## Multivariable logistic regression models

Table S- 8 Multivariable logistic regression models

| **Model** | **Risk Equations** |
| --- | --- |
| **MLR I** | Logit(Risk)= 1.04·10^-1^· MPV+ 2.8·10^-2^· Urea- 4.8·10^-2^· Albumin+ 2.3·10^-2^· Age+ 3.65·10^-1^· Admission type+ 6.45·10^-1^· Heart failure+ 4.44·10^-1^· CKD- 4.467 |
| **MLR II** | Logit(Risk)= 5.4·10^-2^· Urea-5.3·10^-2^· Albumin+ 1.7·10^-2^· Age- 3.727 |
| **MLR III** | Logit(Risk)= -3.3·10^-2^· (CL-) + 3·10^-3^· Creatinine – 5.7·10^-2^· Albumin |

## References

1. Argyropoulos A: **Soft sensor development and process control of anaerobic digestion**. *PhD Thesis*. University of Exeter; 2013.
